# Tracking Scientific Publications in Non-traditional Academic Medical Centers

**DOI:** 10.7759/cureus.103708

**Published:** 2026-02-16

**Authors:** Elli Gourna Paleoudis

**Affiliations:** 1 Medical Sciences, Hackensack Meridian School of Medicine, Nutley, USA; 2 Research Administration, Hackensack Meridian Health Research Institute, Nutley, USA

**Keywords:** institutional publishing, medical publishing, non-traditional academic medical centers, publication tracker, publication tracking, scientific publications, tracking and reporting publications

## Abstract

Tracking academic publications is inherently challenging for both academic and non-traditional academic centers. Publications are considered crucial for academic success; however, accurately identifying and reporting them is a resource-intensive process. There is a lack of a standardized, readily available solution that focuses on tracking publications linked to human subject research within a large healthcare network. This report presents a home-grown approach to tracking and reporting publications. The aim was to track publications and calculate the publication rate of studies approved by the institutional review board (IRB) between 2021 and 2023 at a large United States hospital network by surveying principal investigators (PI) via REDCap about resulting publications. This semi-automated approach revealed a 30.5% publication rate with publications including full-text articles, abstracts, posters, and conference presentations. Tracking publications within a complex healthcare network is challenging. While the described method facilitated data collection, it was time- and resource-intensive, and reliance on PI self-reporting could have limited the accuracy of the publication rate calculated. However, with further automation, home-grown approaches such as the one presented here could allow for a user-friendly application that other non-traditional academic medical centers can adopt. A widely acceptable and easily adaptable tool for publication tracking is needed, particularly for non-traditional academic centers. Future research should explore AI and machine learning applications to address this need.

## Introduction

Publication tracking, a.k.a the systematic search, tracing, and identification of publications from an author or a group of authors, has always been a significant challenge for academia. For decades, publications have been considered the “universal currency” [1] even if the collected and reported metrics, including the number of publications, citation number, and impact factor, have been widely criticized in more recent times [2]. As such, traditional and non-traditional academic centers dedicate significant resources every year to track and accurately report their own publications. Even in the simplest of scenarios, i.e. a smaller-sized academic center with a pre-determined list of potential authors, identifying and accurately reporting the number of publications annually would be a time and labor-intensive process requiring dedicated, often extensively trained resources. The selection of the appropriate search engine(s) is only part of the challenge, with the need to remove duplicates and avoid double-counting and manually reviewing affiliations to avoid mistakenly including authors with the same or similar names, making this task one that even the most experienced librarian would dread. Many of these challenges, including the problem of name disambiguation (identifying authors accurately across different search engines), have been extensively discussed [3], almost introducing a “meta” level in the literature world.

This difficult task becomes almost impossible when we are taking on this responsibility as part of a less traditional academic center. What happens when you do not have a reliable list of faculty to run, or when your institution is more of a network of institutions with multiple different variations of your affiliations? During graduate courses, we are taught about strategies to conduct a systematic literature review or how to formulate a good question such as the PICO (Patient, population, or problem; Intervention; Comparison; Outcome(s))framework [4], its SPIDER (Sample; Phenomenon of Interest; Design; Evaluation; and Research type) adaptation [5] or many others that have been proposed more or less recently. But what happens when these basic principles collapse?

Non-traditional academic centers, including many academic medical centers, have hundreds of potential authors if considering all employees as potential authors, yet realistically speaking, most of these clinicians and others will spend their careers without ever publishing a single scientific article. In these cases, knowing who to include in an advanced search becomes a daunting task.

Variations of the local affiliation can introduce an additional level of complexity. If a network includes many different sites, each with its unique name, authors often will provide as part of their affiliation the name of the site, of the department, of the institution, or any combination of the above. Increase the number of sites from two to three to over 10, and you have a problem that can only be solved with serious coding or a significant amount of manual review.

Last but not least, the rise of open-access journals and pre-print servers amplifies the difficulty of tracking and accurately reporting publications, introducing one more variable, the quality of the publications being sought. Is a publication in a well-recognized, scientific, peer-reviewed journal comparable to a record in a pre-print server?

One would expect that in a world where publications are so important and consequences of inaccurately reported publications could impact significant strategic areas, including but not limited to accreditation, reputation, competitiveness (e.g. for recruitment of faculty, trainees, and students), funding (both institutional and external), academic rank decisions, performance evaluation, and career advancement, a widely accepted solution would have already been identified. That is, however, not the case. This report presents a technical, pragmatic approach to publication tracking and evaluates its feasibility and limitations. More specifically, we describe a home-developed approach to track publications resulting from human subject research studies in a healthcare network comprising 18 hospitals, one medical school, and one basic and translational research center. We discuss lessons learned through the process and conclude with suggestions for other institutions with similar needs.

## Technical report

Methodology

The initial objective of this project was to determine the publication rate for research protocols approved by the Institutional Review Board (IRB) of a large US hospital network, Hackensack Meridian Health, for a pre-determined period of three years (2021-2023) that resulted in at least one publication and generate the relevant “publication rate” (number of studies resulting in a publication/number of studies approved by the IRB). The secondary objective was to establish and document a reproducible methodology for tracking research productivity from IRB approval to final publication, defining the publication rate as the number of studies resulting in a publication divided by the total number of studies approved.

With support from the administrative office of the IRB, a list of approved studies, along with the name of the principal investigator (PI) was generated. REDCap (https://projectredcap.org/resources/citations/), a secure, web-based software platform designed to support data capture for research studies, was utilized to facilitate data collection. Our team partnered with the local REDCap [6-7] administrator to develop a REDCap project where the IRB report was uploaded, and using REDCap features, an automated email was generated and sent to each of the respective PIs (the name and protocol number of the study for which information was requested were listed in the email sent). The email contained a personalized link that directed PIs to a short survey inquiring about a potential publication and the reason why a publication was not achieved, or the type of publication achieved, as applicable. One email reminder was automatically sent to all PIs who did not respond within seven calendar days. PIs with multiple IRB-approved studies within the predetermined period received multiple emails, one per approved study.

For the purpose of this pragmatic approach, a scientific publication was broadly defined as any formal dissemination of research findings that underwent a peer-review process for selection. This includes peer-reviewed full-text articles published or accepted for publication in a scholarly journal, as well as oral presentations and posters delivered at regional, national, or international scientific conferences. Our working definition excludes by default any publications unrelated to the specific study associated with the personalized link, such as publications related to other studies, case reports, opinion pieces, or any other publication resulting from a non-human subject research project. Confirmation of peer-reviewed status took place manually by the author. All articles' citations were cross-referenced with publicly available journal information and confirmed based on PubMed indexing. 

Results

**Figure 1:**
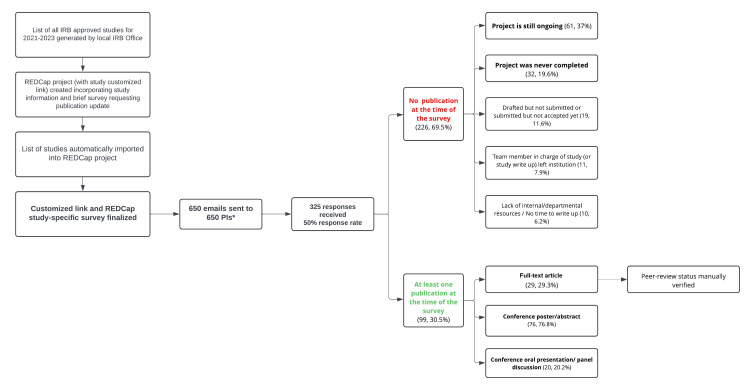
Summary of workflow and survey responses * 650 emails were sent to the 650 PIs of the studies, and PIs that had more than one study received one email per study PI: principal investigator; IRB: institutional review board

The survey remained open for 30 days. A total of 650 IRB-approved studies were identified, and 650 emails were automatically sent to the respective PIs. Within this time, 325 responses were received, resulting in a response rate of 50%. Non-responses included 2% of undeliverable emails, most likely accounting for PIs who left the institution or changed their email address. Of those who responded, 226 (69%) reported that the specific study had not (yet) generated a publication (full-text article/abstract/poster/presentation). The most commonly listed reasons were that the project is still ongoing (n=61, 37%), the project was never completed (n=32, 20%), and the draft is prepared but not submitted, or is prepared and is submitted but has not been accepted at the time of the survey (n=19, 11.6%). Thirty-one (N=99, 31%) PIs reported at least one publication including at least one full-text article (n=29, 30%), at least one abstract/poster (n=76, 77%), and/or at least one conference oral presentation (n=20, 20%). Based on the citations provided, 75% of the full-text articles were published in peer-reviewed journals and were indexed in PubMed. Summary results are presented in Figure 1.

Taking into consideration the 50% response rate, our “publication rate” was 30.5% among responders and 15.2% among all PIs reached through the emails.

## Discussion

The process described was labor-intensive and time-consuming. Although part of the process was automated (initial report, automated emails through REDCap, basic descriptive statistics automatically generated through REDCap), the process still required manual reporting by the PIs and manual verification. Relying on PIs to accurately report publications upon request remains a challenge based on the 50% response rate received. Similarly, response bias might affect response rates, with those who did not publish being less likely to respond. As a result, it is impossible to say whether the 30.5% “Publication rate” reported here actually corresponds to the actual number of publications produced at our healthcare network.

Replicating the process would be feasible, as is further automating the process by creating an API (application programming interface), allowing reports to be automatically fed into REDCap and triggering emails to PIs. However, it has so far been impossible to find a way to circumvent the need for PIs to manually review the information about the requested study and only provide the resulting publication(s) for the respective study.

Reporting on publications for a pre-determined period (e.g., annually) would pose less of a challenge, especially if the list of potential authors is also pre-determined and could be facilitated by the right tool. Several off-the-shelf products promise to automate the capture of affiliated publications, e.g., Esploro (Clarivate Plc, London, United Kingdom) [8], the Elements Platform (Symplectic Ltd., London, United Kingdom) [9], and the open-source ReCiter [10], to name a few, and all of them would potentially facilitate the process if the goal were the simple reporting of publications produced.

Similarly, the more recently introduced Academic Tracker (Samyak Arth Services Private Limited, Varanasi, Uttar Pradesh, India) could also support “publication tracking and reporting by comprehensively searching major peer-reviewed publication tracking web portals, including PubMed, Crossref, ORCID (Open Researcher and Contributor ID), and Google Scholar, given a list of authors” or associated with a specific project and/or grant. Interfolio [11] represents another example, especially for traditional academic institutions, providing tracking tools to faculty and facilitating publication reporting for academic and accreditation purposes. Most of these products are relatively expensive for smaller institutions with limited resources, or can become expensive given the need for customization. In addition, most of these products use an author-based search and can only search publications from a pre-determined list of authors.

For many institutions, it is important not only to understand the publication rate based on the number of studies conducted or the number of studies approved by the IRB, as in the pragmatic approach described here, but also to understand the publication rate from a resource utilization perspective. The need is important enough that many institutions, including ours, take it upon themselves to develop their own more or less successful tools.

## Conclusions

Tracking academic publications is a deceptively complex endeavor. The sheer volume of published research, coupled with variations in author names, institutional affiliations, and even publication venues, creates a tangled web that researchers, librarians, promotion committees, and administrators must navigate. As a result, many institutions choose to report the number of publications as the measure that most accurately represents their impact in the scientific community. Institutions are obliged to develop home-based solutions due to the lack of a well-recognized, universal, and easily adaptable tool. This pragmatic report presents a specific institutional case study. Although the author does not claim generalizability, its purpose is to offer a transparent and reproducible methodology for tracking publication rates in a field where a standardized approach is currently lacking. Market analysis is needed to assess existing products, and more importantly, research is needed to identify the needs of different institutions and leverage AI and machine learning to develop such a tool that would allow institutions to customize their reports based on the ever-changing needs, especially among non-traditional academic centers.
